# A nomogram for predicting risk of death during hospitalization in elderly patients with Alzheimer's disease at the time of admission

**DOI:** 10.3389/fneur.2023.1093154

**Published:** 2023-02-16

**Authors:** Kecheng Yao, Junpeng Wang, Baohua Ma, Ling He, Tianming Zhao, Xiulan Zou, Zean Weng, Rucheng Yao

**Affiliations:** ^1^Department of Geriatrics, The People's Hospital of China Three Gorges University, Yichang, Hubei, China; ^2^Department of Medical Record, The People's Hospital of China Three Gorges University, Yichang, Hubei, China; ^3^Department of General Practice, The People's Hospital of China Three Gorges University, Yichang, Hubei, China; ^4^Department of Respiratory and Critical Care Medicine, The People's Hospital of China Three Gorges University, Yichang, Hubei, China; ^5^Department of Neurology, The First College of Clinical Medical Sciences, Three Gorges University, Yichang, Hubei, China; ^6^Department of Hepatopancreatobilary Surgery, The First College of Clinical Medical Sciences, Three Gorges University, Yichang, Hubei, China

**Keywords:** Alzheimer's disease, comorbidity, in-hospital mortality, nomogram, predictive model

## Abstract

**Background and objectives:**

Elderly patients with Alzheimer's disease (AD) often have multiple underlying disorders that lead to frequent hospital admissions and are associated with adverse outcomes such as in-hospital mortality. The aim of our study was to develop a nomogram to be used at hospital admission for predicting the risk of death in patients with AD during hospitalization.

**Methods:**

We established a prediction model based on a dataset of 328 patients hospitalized with AD -who were admitted and discharged from January 2015 to December 2020. A multivariate logistic regression analysis method combined with a minimum absolute contraction and selection operator regression model was used to establish the prediction model. The identification, calibration, and clinical usefulness of the predictive model were evaluated using the C-index, calibration diagram, and decision curve analysis. Internal validation was evaluated using bootstrapping.

**Results:**

The independent risk factors included in our nomogram were diabetes, coronary heart disease (CHD), heart failure, hypotension, chronic obstructive pulmonary disease (COPD), cerebral infarction, chronic kidney disease (CKD), anemia, activities of daily living (ADL) and systolic blood pressure (SBP). The C-index and AUC of the model were both 0.954 (95% CI: 0.929–0.978), suggesting that the model had accurate discrimination ability and calibration. Internal validation achieved a good C-index of 0.940.

**Conclusion:**

The nomogram including the comorbidities (i.e., diabetes, CHD, heart failure, hypotension, COPD, cerebral infarction, anemia and CKD), ADL and SBP can be conveniently used to facilitate individualized identification of risk of death during hospitalization in patients with AD.

## Introduction

Alzheimer's disease (AD) is the primary cause of dementia and has become one of the main diseases causing death and disability in the elderly population. According to the COAST study, there are 9.2 million dementia cases in China, of which 62.5% are caused by AD (1). It is estimated that by 2050, the number of AD patients will exceed 20 million (2). Compared with elderly individuals without AD, the mortality of patients with AD is 2–4 times higher (3, 4). AD is an independent risk factor for in-hospital death (5), and the 1-year and 5-year mortality rates of patients with AD are higher than those of patients with cardiovascular disease (4). The 1-year mortality of AD patients is 30.5% in women and 38.3% in men (4). Previous studies have noted that comorbidities increase the frequency of hospitalization in community-dwelling persons with AD (6, 7) and contribute to an increased risk of death during hospitalization (8).

Multiple comorbid diseases, including diabetes, cardiovascular disease, cerebrovascular diseases, and chronic kidney disease (CKD), are common in the elderly population, especially in patients with AD (9). Indeed, according to statistics, patients with AD have 2 to 8 comorbidities on average (10). These comorbidities can not only reduce the health-related quality of life but also cause rapid deterioration of clinical conditions that increase risk of death. In addition, with more comorbidities, the related health care costs are higher, and clinicians need to pay additional attention to clinical comorbidity management to help address increasingly tight medical insurance funds (11).

Due to comorbidities, the frequency of repeated hospitalizations in elderly patients with AD has increased significantly, resulting in a meaningful increase in hospitalization mortality, which poses additional challenges to clinical management of AD (12). A 7-year prospective cohort study from the Netherlands examined the incremental value of comorbidity in calculating adverse outcomes for dementia across different predictive periods. The study found that chronic diseases evolve differently over many years and that short-term mortality predictions may be more accurate than those of many prognostic models that focus on several years (12).

A nomogram is a graphical representation of complex mathematical formulas that can reduce statistical predictive models to a single numeric estimate of the probability of a given individual event, such as death or cancer recurrence (13–15). In clinical practice, nomograms are commonly used as risk prediction models for several diseases. Comorbidity factors have been found to successfully predict mortality in a variety of settings, including patients hospitalized in geriatric wards (16, 17). Although studies have reported risk factors for hospitalization death in patients with AD (18, 19), compared with the development of many AD morbidity risk prediction models (20, 21), mortality risk prediction models for hospitalization patients with AD are limited. Consequently, there is a lack of an effective predictive model for hospitalization death risk in this population. The purpose of this study, therefore, was to identify comorbid risk factors associated with mortality and to include these risk factors in the construction of a nomogram for in-hospital death among older people with AD. In this study, we included potential risk factors that may influence mortality risk, such as diabetes, coronary heart disease (CHD), heart failure, hypotension, chronic obstructive pulmonary disease (COPD), cerebral infarction, chronic kidney disease (CKD), anemia, activities of daily living (ADL) and systolic blood pressure (SBP). Our nomogram, an effective and simple prediction tool, may be useful in establishing early warning prognostic systems to evaluate the risk of death during hospitalization by reviewing the medical history of patients with AD at the beginning of hospitalization.

## Methods

### Data source and patient selection

Research approval was obtained from the People's Hospital of China Three Gorges University's Ethics Committee (approval No: PJ-KY2021-26). Patients were retrospectively screened from the China Three Gorges University affiliated People's Hospital from January 2015 to December 2020 and were collected from a hospital-based electronic database. We included subjects who were diagnosed with AD and had comorbidities on admission, a primary diagnosis on admission, and a main diagnoses based on the International Classification of Diseases (9^th^ edition), Clinical modification (ICD-9-CM; WHO 1999) codes (290.0–290.3, 294.1–294.2, and 331.0) and the International Classification of Diseases (10th edition) codes (G30.0–G30.1 and G30.8–G30.9). The enrolled patients had visited in the emergency department or outpatient department due to various clinical manifestations (including fever, cough, chest tightness, chest pain, palpitation, fatigue, edema, abdominal pain, diarrhea, loss of appetite, dizziness, headache, etc.) and were admitted to general wards by the outpatient department or emergency department. The inclusion criteria were as follows: (1) an AD discharge diagnosis and hospitalization for at least 24 h and (2) aged 60 years and over. The exclusion criteria were as follows: (1) basic information could not be obtained (2) vascular dementia, frontotemporal lobar degeneration, Lewy bodies dementia, Huntington's disease and mixed dementia (3) patients admitted to the intensive care unit, and (4) previous medical history information was missing. Demographic, patient comorbidities, and outcome information were collected from electronic medical records.

### Sample size

At present, there is no consensus on how to derive risk prediction models and the method for estimating the sample size needed for validation studies (22). For derivation of models, it is recommended that there should be at least 10 events per candidate variable, and our study included 24 candidate variables in the binary logistic regression model analysis (22). Based on this criteria, the minimum sample size should be 240 individuals.

### Outcome

The risk of all-cause death during hospitalization among patients with AD was the outcome variable of the study.

### Statistical analysis

A total of 328 patients were included in the training set. Statistical analyses were performed using R software (version 3.6.3; https://www.R-project.org) and SPSS version 25.0 (IBM, Armonk, NY, USA). Categorical variables are expressed as frequencies (percentages, %) and continuous variables as means (standard deviations, SDs). Differences in baseline characteristics in the dataset were assessed using Student's *t*-test or the non-parametric Mann–Whitney *U* test for continuous variables and the chi-square test or Fisher's exact test for categorical variables. The “glmnet” package was used to screen factors that may affect the risk of death during hospitalization by the least absolute shrinkage and select operator (LASSO) method; the lambda (λ) with the smallest standard error was selected. The optimal LASSO regression model was constructed, and then the factors with non-zero coefficients selected by the LASSO regression model were included for further analysis (23). LASSO regression models avoid the problems of overfitting and multicollinearity caused by ordinary least squares estimation when there are too many predictors. In this study, univariate logistic regression analysis was used to predict the probability of in-hospital mortality. Multivariate logistic regression analysis was employed to identify independent clinical predictors significantly associated with in-hospital mortality (*P* values <0.05). A nomogram was generated based on the risk factors identified in multivariate analysis. The predictive accuracy of the nomogram was assessed by the area under the receiver operating characteristic curve (AUC-ROC), and calibration curves were calculated to compare predicted probabilities with observed probabilities. Bootstrapping validation (1,000 bootstrap resamples) was performed on the in-hospital mortality nomogram to calculate the corrected C-index. Decision curve analysis (DCA) was applied to determine the clinical usefulness of the in-hospital mortality nomogram by quantifying the net benefit in the AD cohort under different threshold probabilities. The net benefit was calculated by subtracting the proportion of all false-positive patients from the proportion of true-positive patients and weighing the relative harm of abandoning intervention against the negative consequences of unnecessary intervention.

## Results

### Patient characteristics

**Figure 1:**
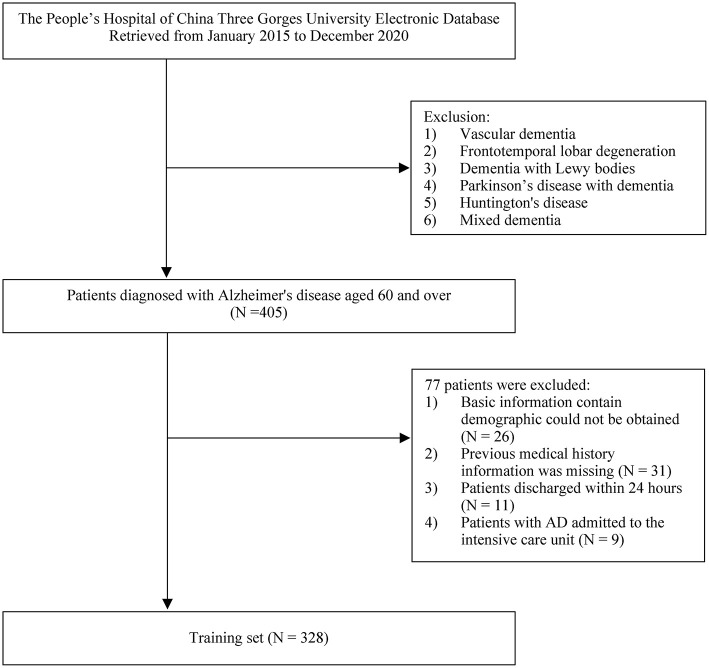
Flow diagram of the selection of eligible patients.

A total of 405 patients with AD were initially enrolled in this study between January 2015 and December 2020. Of these, 77 patients who met the exclusion criteria were removed from the study, and 328 patients were found to be eligible for analysis (Figure 1). The mean time of hospitalization for those patients was 15.46 ± 2.46 days and the average time of AD disease duration was 9.89 ± 3.54 years. The mean score of ADL was 54.42 ± 8.39. In the patients with AD, SBP, and DBP were 130.43 ± 21.02 mmHg and 74.06 ± 11.96 mmHg, respectively. There were 72 in-hospital deaths (22%). The mean age of all patients was 78.03 ± 9.7 years, and the proportions of males and females were 52.1 and 47.9%, respectively. Comorbid diseases included the following: diabetes (ninety patients, 27.4%), hypotension (94 patients, 28.7%), CHD (92 patients, 28%), cerebral infarction (201 patients, 61.3%), heart failure (112 patients, 34.1%), pneumonia (76 patients, 23.2%), CKD (104 patients, 31.7%), COPD (119 patients, 36.3%), and anemia (164 patients, 50.0%). The demographic and disease characteristics of the patients are shown in Table 1.

**Table 1:** Characteristics of patients with AD and in-hospital mortality.

**Characteristics**	**Patients**		**In-hospital mortality**		***P*-value**
	***n***	**%**	***n***	**%**	
Sex					0.902
Male	171	52.1	38	22.2	
Female	157	47.9	34	21.7	
Marital status					0.211
Married	242	73.8	49	20.2	
Other marital statuses	86	26.2	23	26.7	
Age (years)					0.000
<65	31	9.5%	0	0	
65–89	270	82.3%	58	21.5	
≥90	27	8.2%	14	51.9	
Education level (years)					0.048
<9	133	40.5	38	28.6	
9–12	124	37.8	20	16.1	
>12	71	21.6	14	19.7	
AD disease duration (years)					0.004
<3	134	41	22	16.4	
3–10	148	45.3	32	21.6	
>10	45	13.8	18	40	
DM					0.000
Yes	90	72.6	33	36.7	
No	238	27.4	39	16.4	
Hypertension					0.000
Yes	201	61.3	59	29.4	
No	127	38.7	13	10.2	
CHD					0.000
Yes	92	28	47	51.1	
No	236	72	25	10.6	
Cerebral infarction					0.607
Yes	201	61.3	46	22.9	
No	127	38.7	26	20.5	
Heart failure					0.000
Yes	112	34.1	53	47.3	
No	216	65.9	19	8.8	
Hypotension					0.000
Yes	94	28.7	46	48.9	
No	234	71.3	26	11.1	
COPD					0.000
Yes	119	36.3	52	43.7	
No	209	63.7	20	9.6	
Pneumonia					0.000
Yes	76	23.2	31	40.8	
No	252	76.8	41	16.3	
CKD					0.000
Yes	104	31.7	41	39.4	
No	224	68.3	31	13.8	
Anemia					0.000
Yes	164	50	58	35.4	
No	164	50	14	8.5	

AD, Alzheimer's disease; DM, diabetes mellitus; CHD, coronary heart disease; COPD, chronic obstructive pulmonary disease; CKD, chronic kidney disease.

### Variable selection

**Figure 2:**
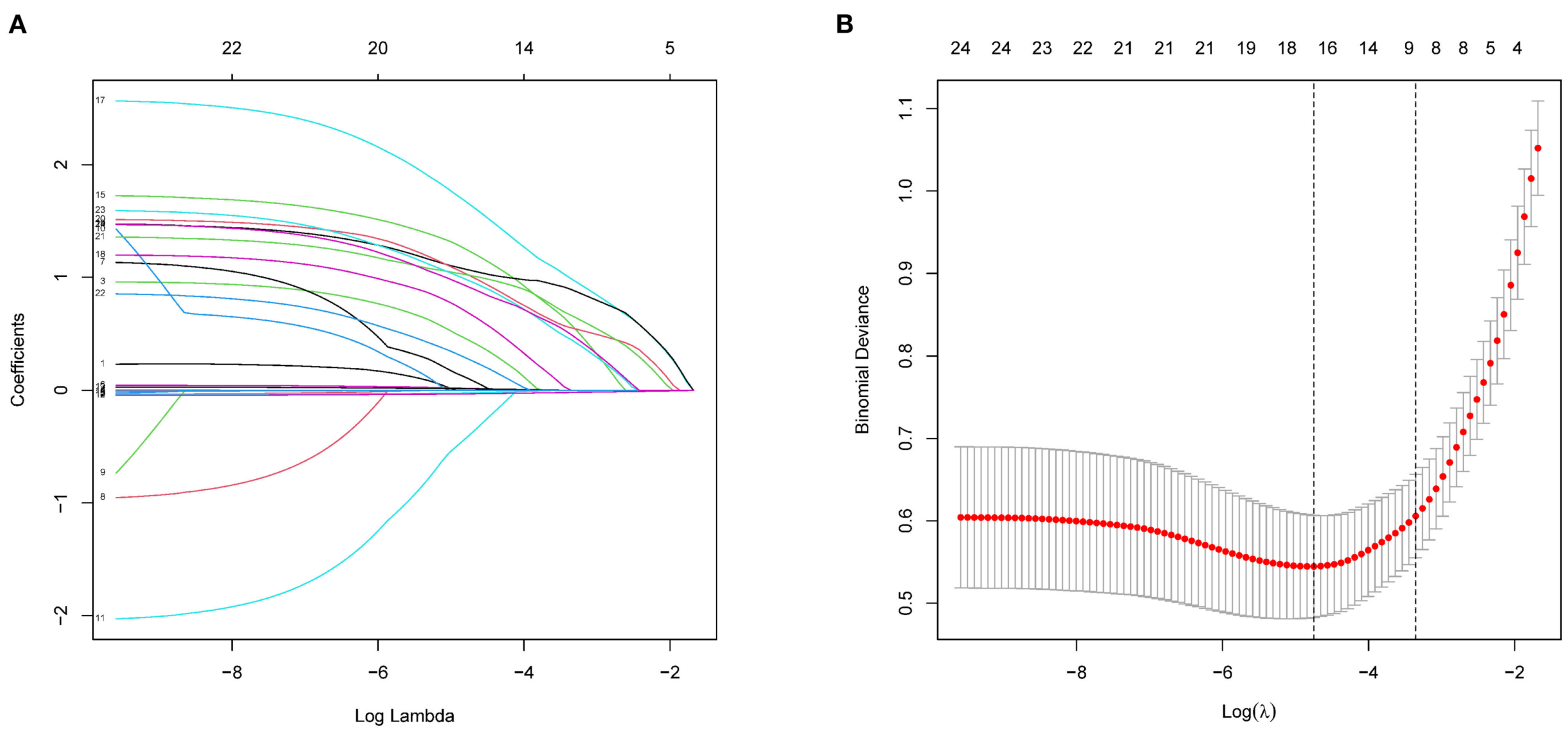
Demographic and clinical feature selection using LASSO binary logistic regression model. **(A)** Five-fold crossover validation of the optimal parameter (lambda) selected in the LASSO model using a minimum standard. Partial probability deviation curves were plotted as compared with log (lambda). A vertical line was drawn at the best using a minimum and a minimum (1-SE) of 1SE. **(B)** A range of LASSO coefficients for 24 characteristics. A coefficient spectrometry was generated based on the logarithm (lambda) sequence. Using fivefold cross-validation, the vertical line was drawn over the selected values, with the best lambda producing 16 non-zero coefficients. LASSO, least absolute shrinkage and selection operator; SE, standard error.

As mentioned, we focused on the effect of comorbidities on death risk in patients with AD during hospitalization at the time of admission. We enrolled patients with known comorbidities and other potential risk factors through history acquisition and included 24 potential risk factors from among these comorbidities and clinical data in LASSO regression analysis (Figure 2). The 24 potential risk factors were reduced to 16 potential predictors that were found to be related to hospitalization mortality with a non-zero coefficient in the LASSO regression model. These 16 features were selected for further analysis and included diabetes, CHD, heart failure, hypotension, COPD, pneumonia, cerebral infarction, CKD, anemia, marriage status, smoke status, in-hospital operation, length of stay (LOS), ADL, AD disease duration, and SBP (Table 2).

**Table 2:** Coefficients and lambda.min value of the LASSO regression.

**Factors**	**Coefficients**	**Lambda.min**
Marriage status	0.409	0.009
Length of stay	−0.023	
AD disease duration	0.014	
Smoke	0.070	
In-hospital operation	−0.344	
ADL	−0.030	
SBP	0.012	
DM	1.201	
CHD	1.606	
Cerebral infarction	0.642	
Heart failure	1.051	
Hypotension	0.987	
COPD	1.007	
Pneumonia	0.245	
CKD	0.944	
Anemia	0.887	

AD, Alzheimer's disease; ADL, activities of daily living; SBP, systolic blood pressure; DM, diabetes mellitus; CHD, coronary heart disease; COPD, chronic obstructive pulmonary disease; CKD, chronic kidney disease.

### Univariate and multivariate analyses

An univariate analysis of the training set revealed that diabetes, hypertension, CHD, heart failure, hypotension, COPD, pneumonia, anemia, CKD, AD disease duration, and SBP were related to the risk of death. These factors were therefore used in multivariate logistic regression analysis for screening independent clinical predictors of hospital death in patients with AD. Multivariate logistic regression analysis demonstrated that 10 variables (diabetes, CHD, heart failure, hypotension, COPD, cerebral infarction, anemia, CKD, ADL, and SBP) were independently associated with hospitalization mortality (*P* < 0.05), as shown in Table 3. These results indicated that these ten variables were independent clinical predictors of hospitalization death in patients with AD.

**Table 3:** Univariable and Multivariate logistic regression analysis for all-cause in-hospital mortality.

**Variable**	**Univariate analysis**			**Multivariate analysis**		
	**OR**	**95% CI**	***P-*** **value**	**OR**	**95% CI**	***P-*** **value**
Marriage status	0.85	0.483–1.504	0.581	2.523	0.859–7.408	0.092
Length of stay	0.971	0.941–1.001	0.062	0.962	0.910–1.017	0.170
AD disease duration	1.045	1.012–1.079	0.007	1.049	0.986–1.116	0.132
Smoke	0.924	0.560–1.522	0.755	1.679	0.641–4.400	0.292
In-hospital operation	0.875	0.384–1.993	0.750	0.250	0.044–1.419	0.117
ADL	0.962	0.952–0.972	0.000	0.958	0.938–0.979	0.000
SBP	1.006	0.994–1.018	0.344	1.025	1.004–1.047	0.021
Diabetes	2.954	1.706–5.116	0.000	6.051	1.942–18.854	0.002
Hypertension	0.274	0.143–0.525	0.000	1.086	0.316–3.740	0.895
CHD	8.815	4.925–15.779	0.000	11.452	3.601–36.424	0.000
Cerebral Infarction	1.153	0.670–1.983	0.607	3.686	1.242–10.943	0.019
Heart Failure	9.314	5.115–16.961	0.000	4.005	1.315–12.197	0.015
Hypotension	7.667	4.317–13.614	0.000	4.625	1.572–13.606	0.005
COPD	7.334	4.081–13.181	0.000	3.119	1.143–8.513	0.026
Pneumonia	3.545	2.011–6.249	0.000	2.177	0.723–6.554	0.167
CKD	4.052	2.346–6.997	0.000	4.171	1.524–11.416	0.005
Anemia	5.863	3.109–11.056	0.000	4.002	1.278–12.526	0.017

Cl, confidence interval; AD, Alzheimer's disease; ADL, activities of daily living; SBP, systolic blood pressure; DM, diabetes mellitus; CHD, coronary heart disease; COPD, chronic obstructive pulmonary disease; CKD, chronic kidney disease. Likelihood ratio test for conditional parameter estimation (forward: Conditional).

### Development of an individualized prediction model

**Figure 3:**
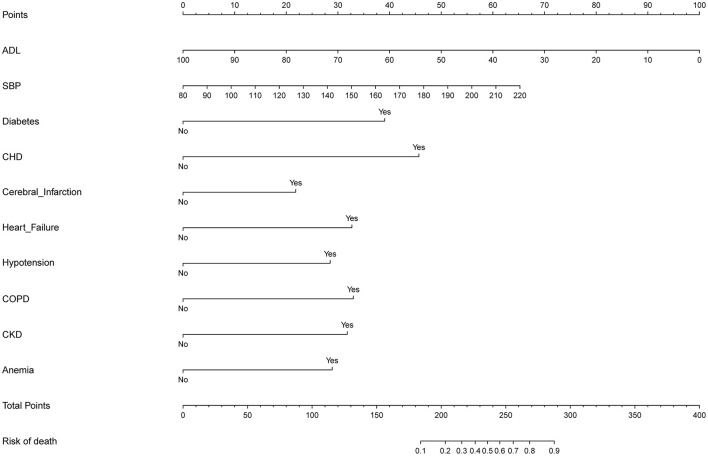
Developed predictor model of in-hospital death nomogram. Nomogram model for predicting individual risk of death in patients with AD. The death risk nomogram was developed in the cohort, with ADL, with SBP, with complications of DM, with complications of CHD, with complications of heart failure, with complications of hypotension, with complications of COPD, with complications of pneumonia, with complications of CKD, and complications of anemia. For all patients adding up the points identified on the point scale for all eight indicators. Then the sum is located on “Total Points” axis. DM, diabetes mellitus; CHD, coronary heart disease; COPD, chronic obstructive pulmonary disease; CKD, chronic kidney disease; ADL, activities of daily living; SBP, systolic blood pressure.

The ten variables selected by logistic regression analysis (diabetes, CHD, heart failure, hypotension, COPD, cerebral infarction, CKD, anemia, ADL, and SBP) were used to build a nomogram for predicting the risk of death during hospitalization in patients with AD (Figure 3). The ratios of the calculated betas were used to evaluate the proportional prognostic effects of these variables. The projections from the total points on the scales below indicated the estimated probability of death during hospitalization.

### Apparent performance of the in-hospital risk of death nomogram in the cohort

**Figure 4:**
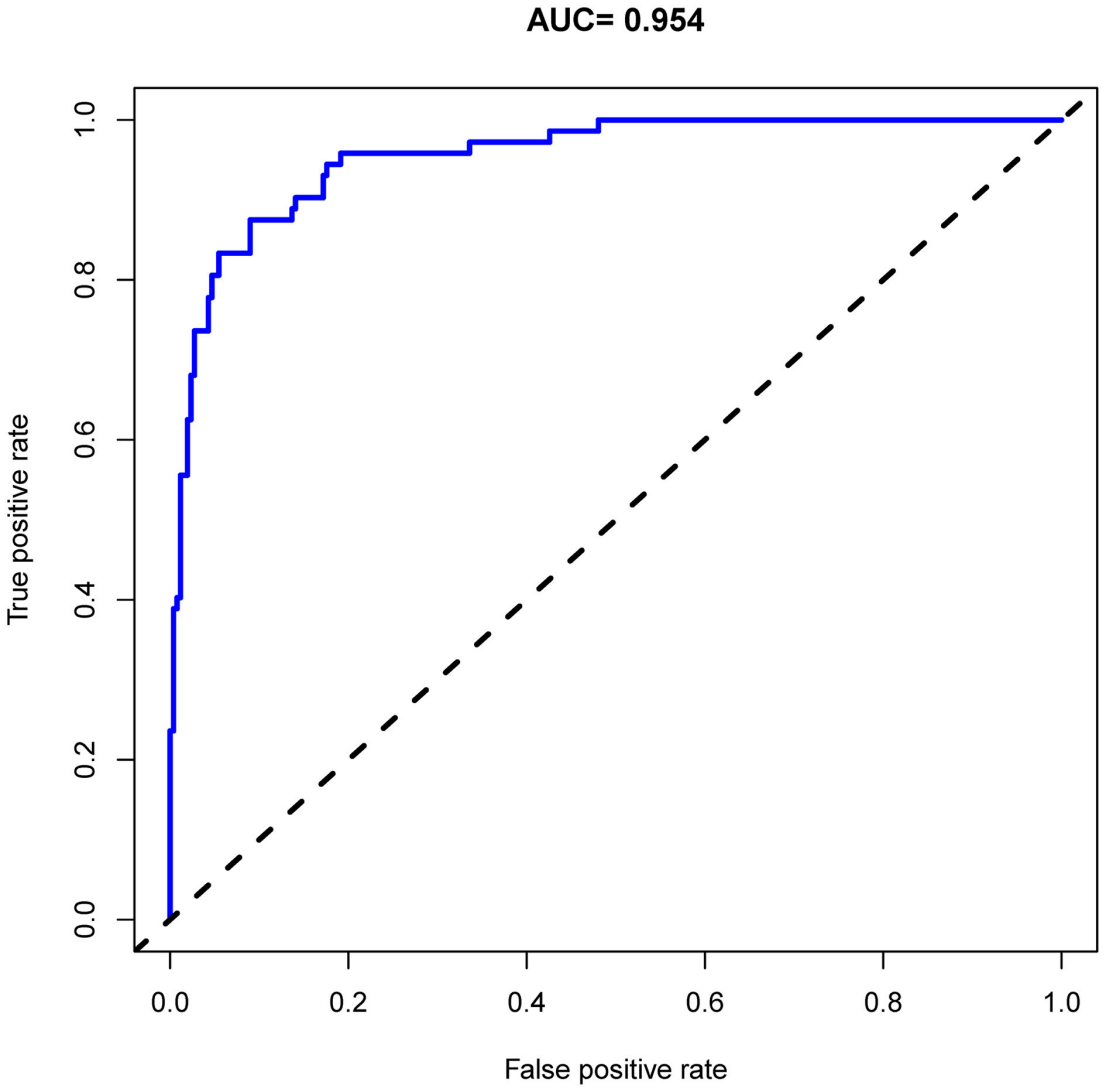
Receiver operating characteristic (ROC) curve analysis of the nomogram for predicting risk of death during hospitalization in patients with AD.

**Figure 5:**
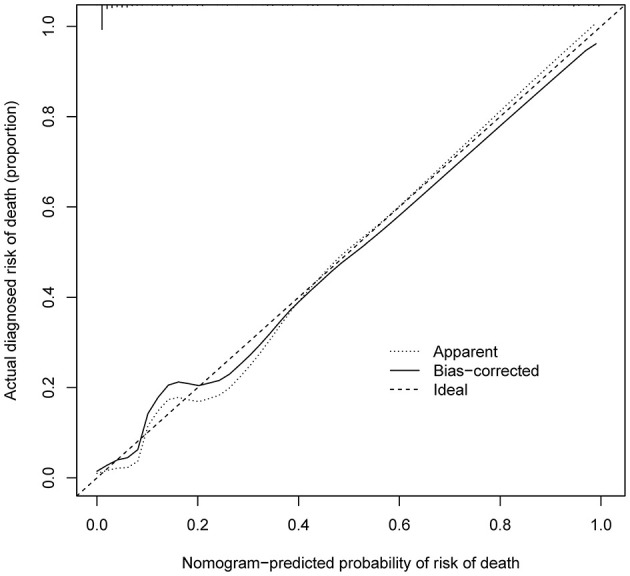
Calibration curves of in-hospital death nomogram predicting in the cohort. B = 1,000 repetitions; boot Mean absolute error = 0.016; Mean squared error = 0.0004. Calibration curve represents probability of patients with AD. The X-axis is the predicted probability by nomogram and the y-axis represents the actual diagnosed risk of in-hospital death. The diagonal dotted line represents a perfect prediction by an ideal model. The black solid line shows the performance of the nomogram, of which a closer fit to the dotted line means a better prediction.

As shown in Figure 4, the model's superior discrimination ability was evident when assessing the performance of the nomogram in predicting death during hospitalization, with the value of the C-index and AUC-ROC obtained using our prediction nomogram being 0.954 (95% CI: 0.929–0.978) in the cohort and confirmed as 0.940 through bootstrapping validation. Figure 5 shows a calibration plot for the dataset. The calibration curve for the model showed excellent concordance between the predicted probability of death during hospitalization and the actual number of observed deaths, with a mean absolute error of 0.016.

### Clinical use

**Figure 6:**
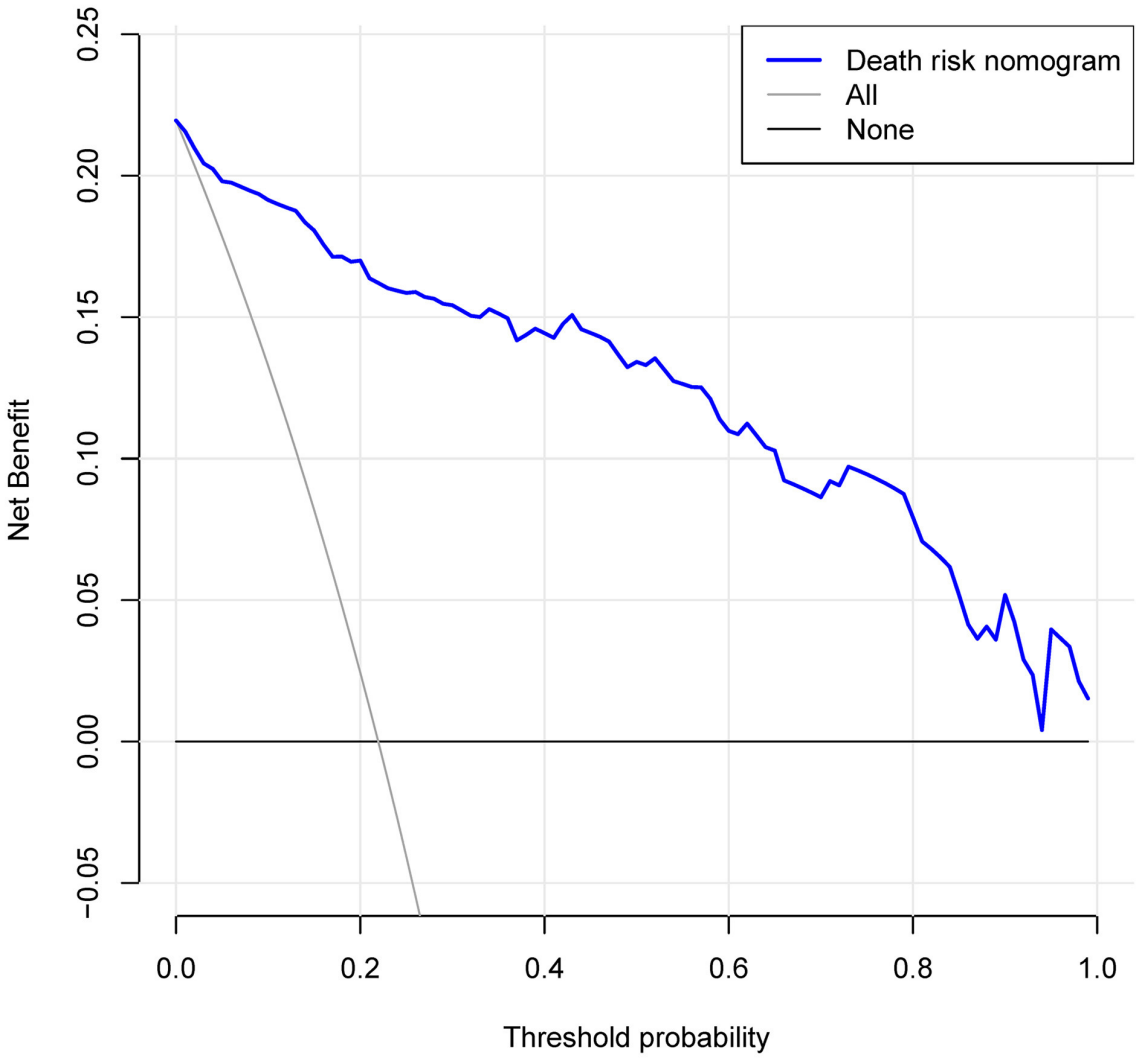
Decision curve analysis for the risk of in-hospital death nomogram. The y-axis measures the net benefit. The dotted line represents the risk of hospital death nomogram. The thin solid line represents the assumption that all patients are left alone. Thin thick solid lines indicate no intervention. The decision curve shows that the use of this nomogram to predict the risk of in-hospital death in this study adds more benefit than patients who do not use this model if the threshold probabilities for patients and doctors are 1 and 99% respectively.

The DCA for the in-hospital death risk nomogram is presented in Figure 6. The results suggested that the threshold probabilities range from 1 to 100% in the dataset and that using this model to predict in-hospital mortality risk adds more benefit. Within this range, the net benefit was comparable, with several overlaps, on the basis of the in-hospital mortality risk nomogram.

## Discussion

In the present study, we developed a nomogram based on comorbid diseases (i.e., diabetes, CHD, heart failure, hypotension, COPD, cerebral infarction, anemia, and CKD), ADL and SBP for use in predicting risk of death during hospitalization in patients with AD. The nomogram demonstrated excellent discrimination and calibration. In addition, DCA results showed a significant net benefit for this model in predicting the risk of in-hospital death for patients with AD.

Patients with AD often have multiple comorbidities, which may be implicated in the pathogenesis and course of AD and play a significant role in its prognosis (24–28). A growing number of clinical and molecular studies have found that chronic diseases such as diabetes mellitus (29) and cardiovascular disease (30, 31) are interconnected. Disruptions in some shared biological pathways is a potential mechanism for the association between AD and these comorbidities (25). For example, some drugs commonly prescribed to patients with diabetes and cardiovascular disease have shown promising results in patients with AD (32–36). In our study, 27.4% of patients had diabetes, 61.3% had hypertension, and 34.1% had heart failure, and these comorbidities accounted for significantly more deaths when compared to those not affected by these diseases. Therefore, it is very important to carefully address the comorbidities in AD and provide personalized treatment (25, 37). Meanwhile, several clinical studies have shown that comorbidity in community-dwelling persons with diagnosed AD affects a wide range of health outcomes, including in-hospital mortality (8, 38). The causes of the adverse outcomes may be related to a lack of attention to pre-existing diseases and inadequate treatment of serious complications that are known to be associated with these diseases (39, 40). Therefore, it is important to consider comorbidities when assessing treatment complications and disease burden. A previous study that followed older people in the community for up to 5 years showed that the activities of daily living and comorbidities were the strongest predictors of mortality risk (17). Although prediction models for individualized risk have been widely used in AD studies, prediction of mortality risk in the hospital setting has not been well documented. In this study, we used data obtained from electronic medical records to evaluate comorbidity and other risk factors associated with in-hospital death of AD patients on admission.

The prognosis of patients with AD requiring admission is generally poor, with in-hospital mortality reportedly at approximately 19.3% (41). In our study, total in-hospital mortality was 22% and was 22.2 and 21.7% for men and women who died during hospitalization, respectively. Because predictive risk models for death in AD patients have not been extensively developed, we generated a prognostic nomogram for AD using individual patient comorbidities during hospitalization. Multivariate logistic regression analysis showed that independent risk factors for in-hospital mortality in AD patients included diabetes, CHD, heart failure, hypotension, COPD, cerebral infarction, CKD, anemia, ADL, and SBP. Using these comorbidities and clinical parameter, we developed a LASSO model with a significant contribution to the risk stratification of AD patients at high risk of death. Overall, this study provides an important innovation in identifying elderly patients at risk of death during hospitalization for AD. It is worth noting that the utility of a predictive model depends on two important criteria: how well the model is calibrated and whether the model can distinguish between high-risk and low-risk patients. Our model meets these two criteria, with a maximum calibration deviation of only 2.5% and acceptable discrimination (AUC = 0.954).

Elderly patients with AD are immunocompromised and often experience complications involving respiratory illness. Prior studies have proven that pneumonia is an independent risk factor for death and is the most commonly identified immediate cause of death among patients with AD (7, 42). Our study found that pneumonia correlates positively with the risk of in-hospital death, but the difference not significant after adjustment by multivariate logistic regression analysis (*P* = 0.167). Our study also found that COPD (OR 3.119, *P* = 0.026) was associated with the risk of death in hospitalized AD patients, which is consistent with findings of a previous study (43). COPD is associated with the development of cognitive deficits, especially with regard to executive function, attention, memory and logical reasoning. In this case, COPD may complicate the management of AD patients; thus, a closer and multidisciplinary monitoring is needed (44).

A cohort study involving 68% of patients with AD showed that the cause of in-hospital death was cardiovascular disease, a risk factor in 23.7% of our patients (5). Our research also found a positive association between cardiovascular disease, comprised of three predictors (CHD, *P* = 0.000), (heart failure, *P* = 0.015), and (hypotension, *P* = 0.005) and in-hospital mortality. Although we evaluated the association between the amount of hypertension in our study population and in-hospital mortality, multivariate logistic analysis showed no significant relationship (*P* = 0.895).

Cerebrovascular disease has become one of the most common comorbidities in patients with AD. With increasing age, cerebral infarction accelerates the rate of cognitive decline, further leading to a series of health care problems due to cognitive dysfunction (26, 45, 46). In our study, the proportion of patients with cerebral infarction was as high as 61.3%, with the proportion of deaths in this group being higher than that found in the non-cerebral infarction patients. It is widely accepted that the severity of cerebral infarction correlates significantly with the prognosis of patients; we also found significant statistical difference (*P* = 0.019) through multivariate logistic regression.

CKD is another common comorbid illness of AD. Renal dysfunction can aggravate cognitive dysfunction in patients. If effective control of CKD is ignored, deterioration of AD patients' wellbeing and quality of life is significantly increased, as is their mortality and the social and family economic burden (47, 48). In our study, patients with CKD accounted for 31.7% of the total cohort, and 25.6% more in-hospital deaths occurred in these patients than in those without CKD. Anemia is a known risk factor for cognitive decline in older adults. One study suggested that assessment of renal function, as well as nutritional and blood status, in older adults diagnosed with AD will help to prevent worsening of CKD and to delay cognitive decline by correcting malnutrition and anemia (49).

Notably, however, not all individuals with a high predicted risk of death will die, and conversely, not all individuals with a low predicted risk of death will survive. Ideally, the predicted risk of death is used to stratify individuals into different groups for allocation of treatment interventions. Our nomogram prediction of prognosis can be used as a guide for selecting suitable patients for intervention, and it can be used jointly with established thresholds for treatment decisions.

### Limitations

The findings of this study must be interpreted in combination with the strengths and possible limitations. A major strength of this study is that it was based on a large number of elderly patients with AD for five consecutive years, such that the outcome was absolutely certain. In addition, as a large university teaching hospital, the research hospital ensures diagnostic accuracy for the AD patients it serves. However, in our retrospective observational study, patients had visited in different ways (emergency or outpatient), resulting in a lack of uniform criteria for assessing individual severity at admission, which is one of the limitations of this study. Therefore, we excluded patients admitted to the intensive care unit after emergency department care to consider the effect of significant differences in treatment and care strategies between the intensive care unit and the general inpatient unit on mortality. Meanwhile, the data used in the analysis were based on administrative data routinely collected by hospitals. Therefore, comorbidities may have been underreported by the patients, which could have led to underestimation of the impact of comorbidities on the risk of death. Another potential weakness is that the predictive model was based on data from a single-center institution; it is necessary to validate our developed model in external datasets and multi-center cohorts. Furthermore, prospective studies are needed to confirm the nomogram's reliability. For example, some patients with AD who are transferred to secondary health care institutions after the treatment phase may be at high risk of in-hospital death. The final model may be biased by misclassifying this small subset of patients as an inpatient survival group when, in fact, some of them may have died at another medical facility.

## Conclusion

In summary, the results of this study suggest that, in addition to ADL and SBP, existing comorbidities, especially cerebral-cardiovascular disease, diabetes, CKD, and COPD, are important risk factors for in-hospital death in patients with AD. The nomogram developed in this study will help clinicians communicate useful prognostic information to help guide treatment decisions.

## Data availability statement

The raw data supporting the conclusions of this article will be made available by the authors, without undue reservation.

## Ethics statement

Written informed consent was obtained from the individual(s) for the publication of any potentially identifiable images or data included in this article.

## Author contributions

KY, XZ, JW, and RY contributed to the study conception and design. Material preparation and data collection was performed by BM, LH, and XZ. Analysis and interpretation of the data were done by KY, ZW, and TZ. The first draft of the manuscript was written by KY, RY, JW, and LH. All authors read and approved the final manuscript.
